# Patterns and influence of familial autoimmunity in pediatric systemic lupus erythematosus

**DOI:** 10.1186/1546-0096-10-22

**Published:** 2012-08-14

**Authors:** Heather M Walters, Nancy Pan, Lakshmi N Moorthy, Mary J Ward, Margaret G Peterson, Thomas J Lehman

**Affiliations:** 1 Komansky Center for Children’s Health/NY Weill Cornell Medical Center, New York, NY, USA; 2 Department of Pediatric Rheumatology, Hospital for Special Surgery, 535 East 70th Street, New York, 10021, NY, USA; 3 Pediatric Rheumatology, Robert Wood Johnson Medical School, University of Medicine and Dentistry of NJ, New Brunswick, NY, USA; 4 Pediatrics, Weill Cornell Medical College, New York, NY, USA; 5 Division of Research, Hospital for Special Surgery, New York, NY, USA

**Keywords:** Pediatric systemic lupus erythematosus, Severity, Inheritance patterns

## Abstract

**Background:**

A high prevalence of autoimmune disease (AD) has been documented in relatives of adult patients with systemic lupus erythematosus (SLE). However, data on familial inheritance patterns in pediatric SLE patients is scarce.

**Findings:**

The charts of 69 patients with pediatric-onset SLE were reviewed retrospectively. The primary aim was to describe the prevalence and types of AD in relatives of children with SLE. The secondary aims were: 1) to compare severity of SLE in children with and without relatives affected by AD, and 2) to evaluate the impact of baseline demographics on severity of SLE in subjects. At diagnosis, 42% of subjects had one or more *first, second, or third degree* relative(s) with AD; and 32% of subjects had one or more *first degree* relative(s) with AD. The most common diseases in relatives of children with SLE were SLE (21%) and thyroid disease (15%). Subjects with no family history of AD were more likely to have severe SLE. SLE severity in subjects did not differ by gender. Children presenting with SLE at an earlier age were found to have more severe disease.

**Conclusions:**

This study demonstrated a high prevalence of AD in families of children with SLE, although a family history of AD did not correlate with more severe SLE in subjects. Future larger studies are necessary to elucidate patterns of familial inheritance and baseline patient characteristics that may affect severity of disease in pediatric SLE.

## Background

Autoimmune disease (AD) affects approximately 5% of the population in the United States [1]. Many studies have demonstrated an increased prevalence of AD in relatives of patients with systemic lupus erythematosus (SLE) and other autoimmune disorders [1-5]. The overall incidence of SLE in the United States is about 1 in 2,000 (0.05%) [6]. Approximately 15% of SLE patients are diagnosed in childhood [7]. Most pediatric studies demonstrate a female to male ratio of two to five females per one male affected with childhood SLE, in contrast to nine females per one male in adult SLE patients [8,9]. Approximately 10% of SLE patients have a relative that also has SLE, and first-degree relatives of SLE patients have an increased probability of having a *non*-SLE autoimmune disease in comparison to the general population [8,9].

Knowledge of familial inheritance patterns in pediatric SLE and influence upon SLE severity in subjects is limited. The primary aim of this study was to describe the prevalence and types of AD occurring in families of children with SLE. The secondary aims were: 1) to compare severity of SLE in children with and without relatives affected by AD, and 2) to evaluate the impact of baseline demographics on severity of SLE in subjects.

## Methods

A systematic retrospective chart review was conducted at a pediatric rheumatology clinic at the Hospital for Special Surgery. All study work was conducted in accordance with the requirements of the Helsinki Declaration, and this study was approved by the Institutional Review Board at the Hospital for Special Surgery.

69 patients who fulfilled SLE diagnostic criteria (as established by the American College of Rheumatology) and had onset of disease prior to age eighteen years were included [10]. Patients included were followed between the years 1990 and 2010. Data collected included age at diagnosis, gender, ethnicity, family history of AD at diagnosis, and treatment course for SLE in subjects.

Severity of SLE in subjects was inferred by the type of treatment required to control disease. Subjects requiring no chronic immunosuppressive therapy were considered to have mild SLE. Subjects requiring chronic steroids only were classified as having moderate SLE. Subjects requiring therapy with cyclophosphamide, rituximab, mycophenolate mofetil, and/or azathioprine were classified as having severe SLE.

Univariate and multivariate analyses were conducted. Chi-square, Mann-Whitney, and ANOVA or t-tests were used as appropriate. SPSS software was utilized, and results were considered statistically significant at p < 0.05.

## Findings

57 females (83%) and 12 males (17%) were included. 25 subjects were Latino (36%), 17 were Asian (25%), 16 were Caucasian (23%), 10 were African American (15%), and one ethnicity was unknown (1%). As defined by treatment courses required, 6 subjects (9%) had mild SLE, 17 subjects (24%) had moderate SLE, and 46 subjects (67%) had severe SLE. Disease duration ranged from one to twenty years. Organ involvement was distributed as follows: renal involvement in 37 patients (54%), skin involvement in 27 patients (39%), arthritis in 25 patients (36%), hematologic involvement in 24 patients (35%), neurologic involvement in 9 patients (13%), and cardiopulmonary involvement in 3 patients (4%).

**Figure 1:**
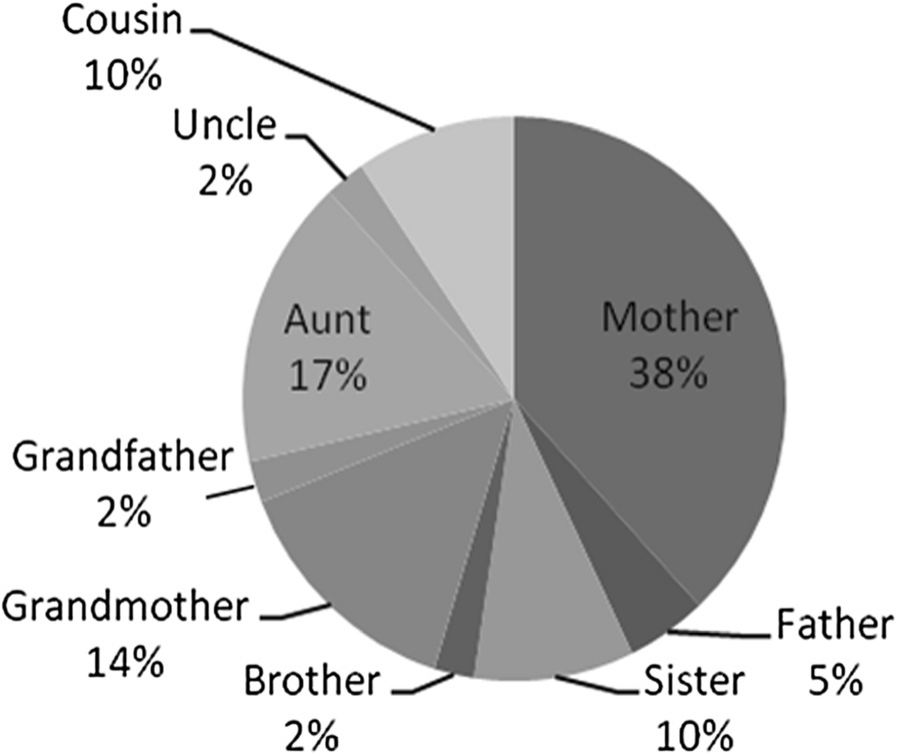
Relatives of pediatric SLE patients affected by autoimmune disease.

62 patients had complete documentation of family history. 26 (42%) had one or more *first, second, or third degree* relatives with AD. Mothers were most commonly affected (38%), followed by aunts (17%), and grandmothers (14%). See Figure 1. 20 subjects (32%) had one or more *first degree* relative with AD. Mothers were again most commonly affected (70%), followed by sisters (17%).

The most common AD in relatives of subjects was SLE. 13 subjects (21%) had a *first, second, or third degree* relative with SLE, and seven subjects (11%) had a *first* degree relative with SLE. The second most common AD in relatives of subjects was unspecified thyroid disease. Nine subjects (15%) had a *first, second, or third degree* relative with thyroid disease, and seven subjects (11%) had a *first* degree relative with thyroid disease. See Table 1 for the prevalence of each noted AD in relatives of subjects. See Table 2 for the categorization of relatives affected by a specific AD.

**Table 1:** Number of pediatric SLE subjects with relatives affected by specific ADs

**Type of AD**	**Subjects with any relative (N = 62)**	**Subjects with 1**^**st**^**degree relative (N = 62)**	**Subjects with 2**^**nd**^**degree relative (N = 62) **	**Subjects with 3**^**rd**^**degree relative (N = 62)**
SLE	13 (21%)	7 (11%)	5 (8%)	5 (8%)
Thyroid	9 (15%)	7 (11%)	3 (5%)	0
Psoriasis	4 (6%)	3 (5%)	1 (2%)	0
MCTD	3 (5%)	2 (3%)	1 (2%)	0
RA	3 (5%)	1 (2%)	2 (3%)	0
MS	2 (3%)	1 (2%)	1 (2%)	0
SA	1 (2%)	1 (2%)	0	0
Vitiligo	1 (2%)	1 (2%)	0	0
IDDM	1 (2%)	0	1 (2%)	0

*N number of subjects with a complete family history available.

*Thyroid thyroid disease, *MCTD* mixed connective tissue disease, *RA* rheumatoid arthritis, *MS* multiple sclerosis, *SA* spondyloarthropathy, *IDDM* insulin-dependent diabetes mellitus.

**Table 2:** Categorization of relatives of pediatric SLE subjects affected by specific ADs

**Relative with AD**	**SLE (n = 18)**	**Thyroid (n = 10)**	**Psoriasis (n = 4)**	**MCTD (n = 3)**	**RA (n = 5)**	**MS (n = 2)**	**SA (n = 1)**	**Vitiligo (n = 1)**	**IDDM (n = 1)**
Mother	5	7	1	1	1	1	1	0	0
Father	0	0	2	0	0	0	0	0	0
Sister	2	0	0	1	0	0	0	1	0
Brother	1	0	0	0	0	0	0	0	0
Grandmother	1	2	1	0	2	0	0	0	0
Aunt	4	1	0	1	0	1	0	0	0
Uncle	0	0	0	0	2	0	0	0	1
Cousin	5	0	0	0	0	0	0	0	0

*n = number of relatives of subjects affected by a specific AD.

*Thyroid = thyroid disease, *MCTD* = mixed connective tissue disease, *RA* = rheumatoid arthritis, *MS* = multiple sclerosis, *SA* = spondyloarthropathy, *IDDM* = insulin-dependent diabetes mellitus.

Subjects with a family history of AD were more likely to have mild or moderate SLE, and subjects with no family history of AD were more likely to have severe SLE. 70% of patients with mild or moderate SLE had a *first, second, or third* degree relative with AD, while only 29% or patients with severe SLE had a *first, second, or third* degree relative with AD (p < 0.05). Additionally, 60% of subjects with mild or moderate SLE had a *first degree* relative with AD, while only 19% of subjects with severe SLE had a *first degree* relative with AD (p < 0.01).

Severity of disease did not appear to differ by gender (p = 0.62). Subjects with severe disease had an earlier average age of onset than those with mild disease (12 versus 16 years, p < 0.05).

## Discussion

This cohort of subjects seems to accurately represent pediatric SLE patients overall, with a female predominance of 83%, and a female to male ratio of approximately five to one [8,9]. Most patients in this cohort had severe SLE (67%), as defined by treatment course required. This was likely influenced by the selection of patients from a tertiary care center.

The children with SLE in this study had a high prevalence of family members with AD. However, the prevalence of familial AD in our subjects (42%) was lower than some previous studies, which have shown up to 74% of subjects with AD also have a family history of AD [3]. As suggested in many prior studies, we can therefore conclude that genetics clearly contribute to the development of pediatric SLE, but other factors influence the evolution of this disease as well. An environmental trigger may be necessary to produce clinical disease in a genetically-susceptible host. The lower prevalence of familial AD in this study is likely due to other factors contributing to the development of SLE which are not yet elucidated.

In this study, the most common AD in relatives of pediatric SLE subjects was also SLE (21%). This rate is higher than previously quoted rates of approximately 10% of SLE patients having a family history of SLE [8]. As our study focused upon pediatric SLE patients, our findings may indicate that pediatric patients with a family history of SLE are more likely to be diagnosed in childhood when presenting with SLE-like symptoms. Patients diagnosed with SLE in adulthood may be less likely to have a family history of SLE, leading to later consideration of this disease in a diagnostic evaluation. As documented in previous studies, thyroid disease was also common in relatives of our pediatric subjects with SLE (15%), likely because thyroid disease is a relatively common autoimmune disease in the general population [3,5,9].

In this cohort, mothers of subjects were the most commonly affected relative (38%), followed by aunts (17%), and grandmothers (14%). A higher prevalence of affected female relatives is consistent with the known higher prevalence of females with AD in the general population [8]. However, the high prevalence of maternal family history may be influenced by recall bias, as mothers accompanying a pediatric patient may be more likely to report a personal medical history of AD, and may sometimes be unaware of extended family history of AD.

A family history of AD did not predict more severe SLE in patients in this study. In fact, patients with a family history of AD were more likely to have mild or moderate SLE, and patients with no family history of AD were more likely to have severe SLE. As suggested above, perhaps this is because patients with a known family history of AD are more likely to receive a full diagnostic work-up for SLE when presenting with symptoms that may be autoimmune in origin, even when these symptoms are mild. In contrast, a patient who presents with obvious symptoms of severe SLE is likely to be evaluated for SLE regardless of family history.

Subjects with more severe disease presented with SLE at an earlier age in this cohort. Prior studies have also demonstrated a more severe phenotype in pediatric-onset SLE patients than in adult-onset patients (higher risk of proteinuria, cellular casts, arthritis, hemolytic anemia, *etc.*) [11]. This may be because the first clinical manifestations of SLE become evident earlier in patients predisposed to develop a severe SLE phenotype.

Our study had several limitations, including a small sample size and a retrospective approach. Additionally, several incomplete charts led to a lack of comprehensive family history documentation for several subjects.

This study demonstrates a high prevalence of AD in families of children with SLE. However, a family history of AD did not seem to correlate with a severe disease phenotype in pediatric SLE subjects. Improved characterization of familial inheritance patterns may enhance our ability to diagnose patients with AD earlier, leading to prompt treatment and improved outcomes for pediatric patients. These findings highlight the need for future larger studies, conducted across generations, to further elucidate patterns of inheritance and factors influencing severity of disease in pediatric SLE and other autoimmune diseases.

## Abbreviations

AD: Autoimmune disease; SLE: Systemic lupus erythematosus.

## Competing interests

Heather M Walters, Nancy Pan, Lakshmi N Moorthy, Mary J Ward, Margaret G Peterson, Thomas J Lehman declare that they have no competing interests

## Authors contributions

HMW contributed to the study design, data collection, data analysis, review of results, and manuscript preparation. NP contributed to the data collection. LNM contributed to the study design, data analysis, review of results, and manuscript preparation. MJW contributed to data analysis. MGEP contributed to data analysis. TJAL contributed to the study design, review of results and manuscript preparation. All authors read and approved the final manuscript.
